# Applicability of UV-Curable Binders in High Solid Suspensions for Direct-Ink-Write 3D Printing in Extremely Cold Temperatures

**DOI:** 10.1021/acsami.3c11742

**Published:** 2023-10-20

**Authors:** Alexandra Marnot, Lena Konzelman, Jennifer M. Jones, Curtis Hill, Blair Brettmann

**Affiliations:** † School of Chemical and Biomolecular Engineering, Georgia Institute of Technology, Atlanta, Georgia 30332, United States; ‡ George W. Woodruff School of Mechanical Engineering, Georgia Institute of Technology, Atlanta, Georgia 30332, United States; § NASA Marshall Space Flight Center, Huntsville, Alabama 35898, United States; ∥ NASA Marshall Space Flight Center, Jacobs Space Exploration Group, Huntsville, Alabama 35898, United States; ⊥ School of Materials Science and Engineering, Georgia Institute of Technology, Atlanta, Georgia 30332, United States

**Keywords:** material extrusion, thermal extremes, high solid suspensions, UV curing, cross-linking

## Abstract

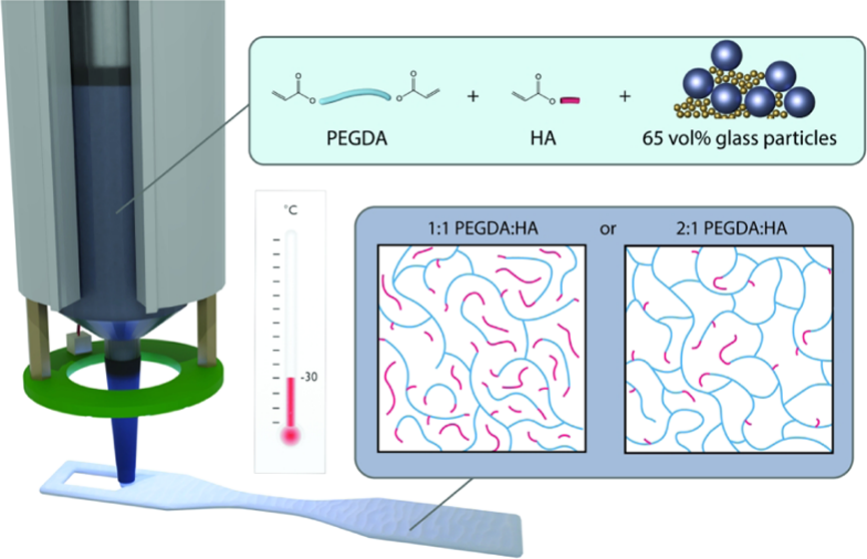

Leveraging material extrusion 3D printing of high solid suspensions for rapid manufacturing in future space missions requires materials compatible with the unique environments found on the Lunar surface. However, there is currently a lack of selection criteria for materials processable in the harsh environmental conditions on the Moon without significantly altering the 3D printers. Here, we provide valuable insights into the behavior of high solid suspensions at low temperatures to guide informed decision-making for manufacturing in subzero environments. We investigate the effects of direct-ink-write (DIW) printing at −30 °C on the structure–property relationships of UV-curable high solid inks of glass microspheres. We analyze the inks based on extrudability and curability at subzero temperatures to verify extrusion, shape retention, and sufficient solidification, culminating in successful printing at −30 °C. Preferential polymerization among monomers is observed at −30 °C and results in a lower cross-linking density in the final print, with a reduced tensile modulus. However, lower ratios of highly mobile monomers result in the retention of mechanical properties, demonstrating the selection criteria for binder design. Through this work, we highlight the importance of binder formulations used for 3D printing in uncommon environmental conditions that are emerging as tomorrow’s manufacturing challenge.

## 1 Introduction

High solid suspensions, those containing more than 50 vol % solids, have become increasingly common feedstocks for additive manufacturing (AM) methods due to the need for the functionality of the solid phase and the precise control, rapid prototyping, and ink design space provided by AM.^1−3^ Direct-ink-write (DIW), a type of material extrusion AM, in particular, has been demonstrated for processing a variety of highly loaded suspensions in fields ranging from electronics, aerospace, regenerative medicine, and construction.^4−11^ In DIW of high solid suspensions, solids are typically suspended in a liquid binder, extruded through piston, pneumatic, or screw-driven displacement, and a solidification step is imparted on the binder phase to harden the extrudate.^12−14^ The appeal of DIW for high solid suspensions is the relatively large formulation design parameter space able to be used with this setup, in part driven by the many modes of ink solidification mechanisms, including solvent evaporation, light curing, and thermal curing. This makes the technique flexible if the solid phase is sensitive to a particular stimulus. For instance, some solids used in the medical sector are sensitive to heat, and therefore, evaporation or light-curing are more favorable solidification mechanisms than thermal cure.^15^

While a great focus has been dedicated to binder formulation design to provide the most optimal printing conditions for the suspensions,^16−18^ recent progress in space exploration efforts has triggered the need for more high-performing materials and for the applicability of 3D printing methods in isolated and demanding environments such as the Lunar surface.^19−21^ Now, not only must binder formulations be designed to optimally process various kinds of solid materials, but they must also be tailored for printing conditions including microgravity, vacuum, thermal extremes, solar and cosmic radiation, as well as dust storms and charging.^22−25^ Experiments conducted on the International Space Station by Made In Space, Inc. demonstrated the first success of 3D printing in microgravity by fused filament fabrication (FFF).^26^ For DIW, the technique was used in microgravity by Li et al. to deposit droplets of a very dilute colloidal suspension and through Techshot’s BioFabrication Facility on the International Space Station, and work by Leu and co-workers demonstrated a very good use case of DIW in cold temperatures with freeze-form extrusion fabrication (FEF).^27,28^ Since then, other demonstrations of FEF in cold temperatures have been employed for a variety of formulations, including some with high solid loadings, with subzero temperatures applied to either the print bed, the ambient atmosphere, or both.^29−32^

Nevertheless, most FEF demonstrations rely on the extreme thermal difference to provide the necessary rigidity to extruded inks, mostly by means of freezing an aqueous suspension, and the binder is often removed following the completion of the printing cycle.^28,32^ To our current knowledge, no DIW of highly loaded suspensions has been demonstrated in an environment with extreme thermal conditions with the intention of replicating an existing room temperature process at subzero temperatures rather than utilizing subzero temperatures as the solidification mechanism. As such, the differences that may arise from attempting to carry out a well-studied ambient DIW printing process at temperatures well below 0 °C are not well understood. There is, thus, a need to leverage the formulation design space available for DIW inks with regard to successful printing in harsh environmental conditions.

The forefront challenge in DIW of high solid suspensions is ensuring extrudability despite the high fraction of solids, resulting in more frictional contacts between particles and an increased potential for jamming in the printer nozzle. Continuous extrusion is a requirement for printed parts that are mechanically sound, and therefore, DIW inks should flow homogeneously for the entirety of the print volume. Formulation design strategies that can help ensure extrudability include using a multimodal distribution of the solid particles, the use of carefully selected binders to maintain suspension homogeneity, and viscosity modifiers such as surfactants or reactive diluents to assist with flow.^10,33−35^ With regards to particle size modality, lowering the printing environment temperature below 0 °C should result in minimal changes to the suspension’s extrudable properties if the particles are noncolloidal and Brownian motion is not significant.^36^ However, the same is not necessarily true for binders and additives, whose physical and chemical properties can be very temperature-dependent. Tailoring the binder formulation of high solid DIW inks typically requires addressing two criteria: lubricating contacts between particles to reduce friction and ensure flow while cohesively keeping the distribution of particles homogeneous throughout the suspension volume, and undergoing a rapid solidification step post-extrusion. Subzero temperatures present an additional challenge to meeting these criteria, as temperature-induced phase changes, with a rapid increase of the binder viscosity, can easily result in a suspension that is too viscous to extrude. The FEF technique, for example, can suffer from nozzle clogging due to the freezing front progressing into the extruding nozzle.^37^ Additionally, thermal effects can significantly impact the solidification of the binder post-extrusion, whether by causing the solvent to freeze rather than evaporate or by causing thermal gradients and uneven cure along the printed layers.^37^

Attempting to generate a binder for a DIW ink to be used in a simulated environment representing the Lunar surface therefore means assessing different solidification mechanisms with regards to environmental conditions like thermal extremes while ensuring extrudability of a high fraction of solid particles.^24^ UV cure is a viable method for solidifying inks post-extrusion despite cold temperatures. Here, a mixture of monomers and oligomers can be tailored to achieve a particular viscosity and reactivity, and the polymerization reaction is not as impeded by temperature extremes as thermal curing and chemical reactions between the solids and the binder.^7,38^ However, a challenge arises with selecting UV curing as the solidification mechanism for printed inks with a high particle loading since a certain dosage of UV light is required to initiate the polymerization reaction of the binder components. This dosage must be supplied at sufficient depths to cure individual layer heights despite the high content of solid particles scattering, reflecting, and absorbing some of the UV light,^38^ and the curability of the binder therefore becomes another major criterion for the design of the DIW ink binder. When considering multimodal ratios of particle sizes to assist with extrudability, smaller particles result in even greater challenges to light penetration due to their increased scattering efficiency.^39^ As such, most AM applications that use small, absorbing, and opaque particles, such as in stereolithography (SLA) and in digital light processing (DLP), maintain suspension loadings below 50 vol %.^40−42^ DIW, with its ability to enable taller layer heights and use inks formulated with larger particles,^19,42,43^ offers advantages over SLA and DLP in terms of printing efficiency and UV curing of larger particles with lower potential for light scattering.^41^ Provided that the formulation design of the binder meets both the extrudability and curability criteria, this technique could be deployed for rapid manufacturing in environments with very harsh thermal extremes.

The objective of this study is therefore 2-fold: understanding the effect of a subzero temperature on the rheology and solidification properties of high solid suspensions processed by DIW and demonstrating a successful formulation candidate for printing parts containing 65 vol % of solid particles at −30 °C. We chose to utilize soda-lime glass microspheres as a generalizable case of solid particles in a bimodal ratio established from our previous work that would allow a high loading of particles without resulting in discontinuous extrusion.^44^ Our binder system was selected to consist of a short acrylate monomer and a larger prepolymer acrylate to also investigate the effect of temperature on interactions and bonding between the two components. Printing comparisons are established between ambient (25 ± 2 °C) and −30 °C, and the rheology and curing kinetics of the inks are assessed for both cases. We then tie the environment-affected ink microstructure to the resulting mechanical properties of the printed parts. With this work, we aim to facilitate greater use of UV-assisted DIW and specifically to widen the knowledge base of processing–property relationships for high solid suspensions used in the extreme and demanding environments that will be encountered during future space exploration missions.

## 2 Results and Discussion

**Figure 1:**
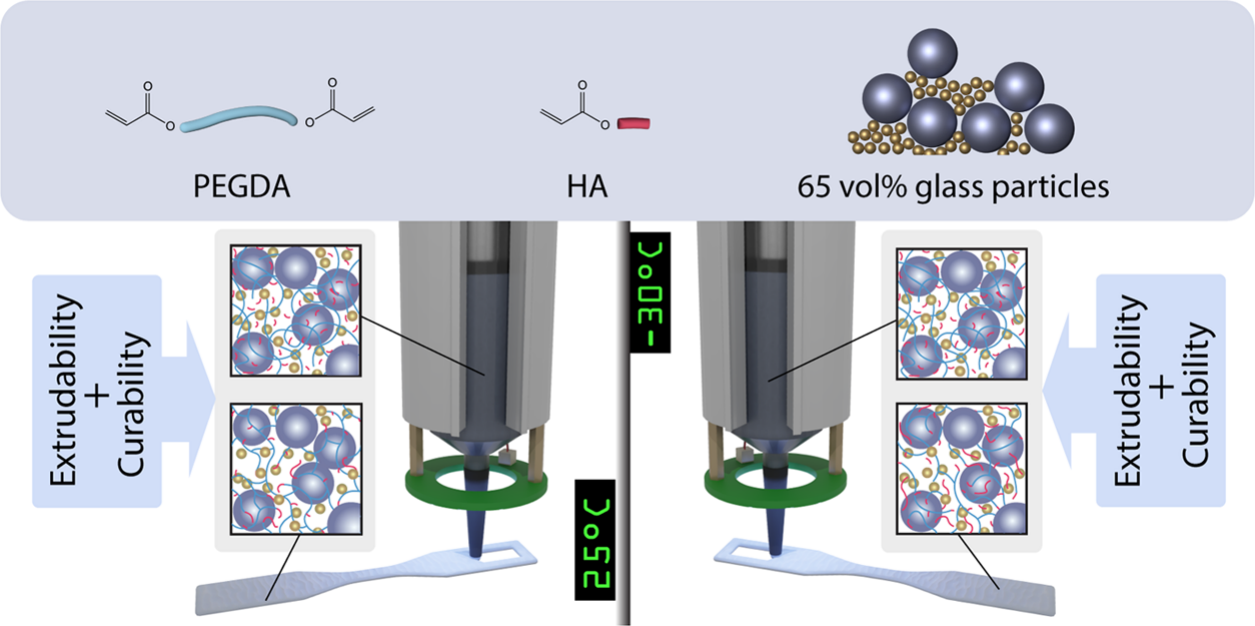
Summary of the mirror printing process with a knowledge base of structure and processing requirements established and verified at 25 °C and verified for processing in extreme cold at −30 °C.

To extend the capabilities of additive manufacturing and effectively utilize UV-assisted DIW in subzero temperatures, we undertook a formulation-based approach to develop inks capable of being processed in demanding environments with minimal modifications to the printing setup. We leveraged the apparent extrudability and curability criteria requirements, with respect to their performance in response to low temperatures, to facilitate the design selection for the binder formulation of the DIW ink (Figure 1). Inks were, therefore, initially evaluated for printability at room temperature, as per the extrudability and curability criteria, which demonstrated continuous extrusion, good shape fidelity, and sufficient levels of cure to forego an intralayer curing step. Printing was also achieved at −30 °C in a mirror process of ambient printing and required local heating of the barrel and nozzle to maintain extrudability. Results also indicate that selecting binder components with an emphasis on curability plays a strong role in determining the mechanical properties of prints since −30 °C temperature affects the binder’s cross-linked microstructure. The formulation optimization for the materials studied here is less dependent on the extrudability criteria as it only needs to be maintained with heating. By prioritizing a consistent cross-linked microstructure at cold temperatures, a diverse range of DIW formulations with a variety of particle types can be created for further DIW printing in harsh environmental conditions.

### 2.1 Extrudability

Toward ensuring extrudability, suspensions with more than 50 vol % solid particles are a challenge. This is due to an increased potential for jamming stemming from more frictional contacts and can be verified by assessing the suspension viscosity. At our chosen solid loading of 65 vol %, high viscosities can result in significant nozzle clogging and failed extrusion. To minimize this potential outcome, we utilized a bimodal particle size distribution of spherical soda-lime glass particles to optimize solid packing (for the large particles: *d*_10_ = 235 μm, *d*_50_ = 271 μm, *d*_90_ = 317 μm, and for the small particles: *d*_10_ = 10 μm, *d*_50_ = 35 μm, *d*_90_ = 80 μm). We also used a low-viscosity binder composed of a difunctional acrylate polymer, poly(ethylene glycol) diacrylate (PEGDA) and hexyl acrylate (HA), a monofunctional monomer, and a reactive diluent. We tested two ratios of PEGDA:HA, 1:1 and 2:1 vol/vol for the binder, in an effort to elucidate how the proportions of longer prepolymers with repeating units (PEGDA) and single-unit monomers (HA) would affect the viscosity of the ink and, by extension, its extrudability. While these formulation design choices are favorable for meeting the extrudability criteria in or near ambient environmental conditions (see the continuous shear-thinning profiles in Figure S1), there is little understanding of how to select monomers as binder materials or what monomer ratio to use for extruding 65 vol % particles at −30 °C. To this end, we further justified the use of PEGDA and HA for their low glass transition temperatures (−39 and −57 °C, respectively) and relatively low viscosities at 25 °C (0.01 and 0.1 Pa·s, respectively), which were hypothesized to allow for processability at −30 °C, due to a certain level of retained molecular mobility.

**Figure 2:**
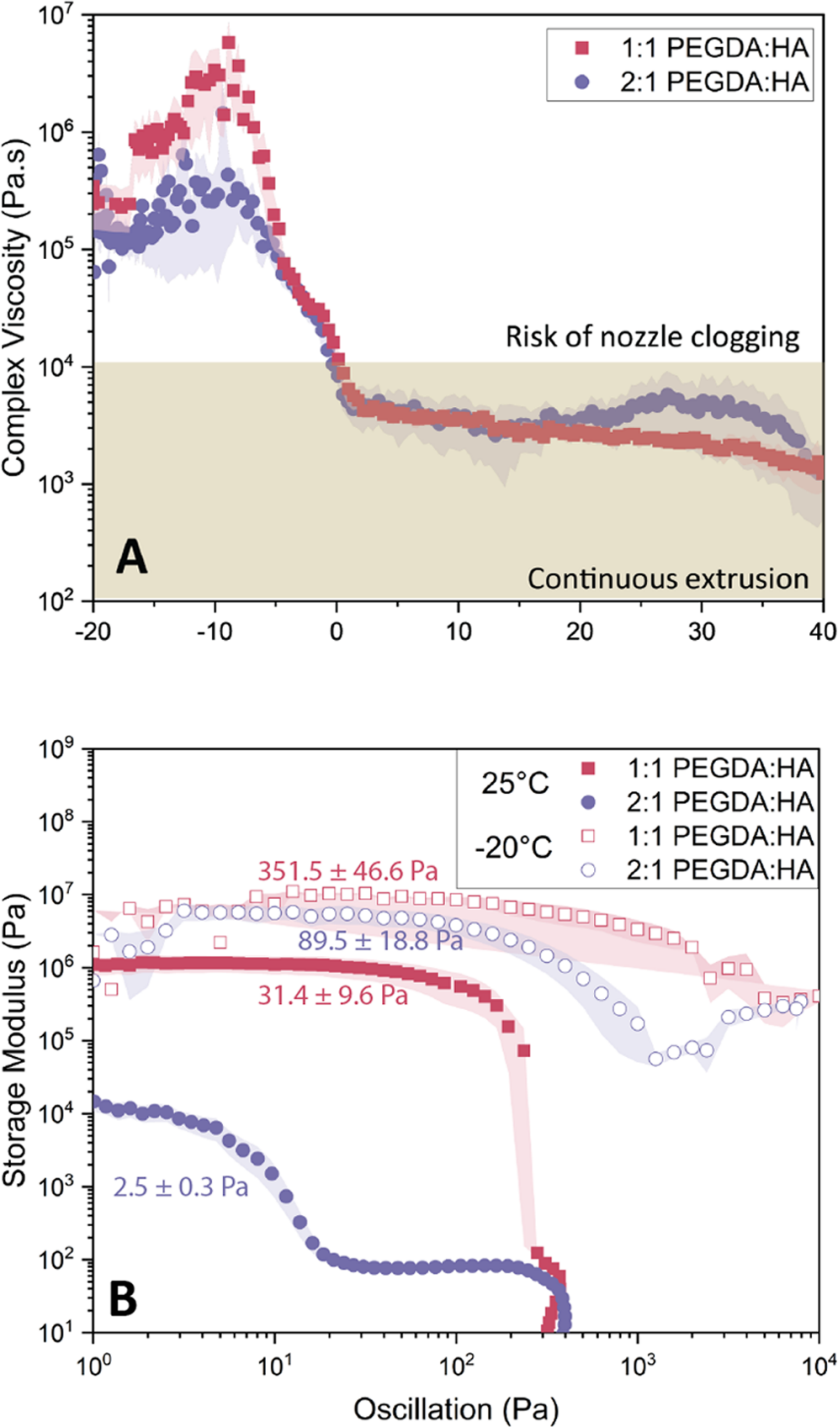
(A) Viscosity evolution with temperature for uncured inks containing 65 vol % glass microspheres. Regions of continuous extrusion and potential for nozzle clogging follow observed trends of extrusion quality, which are available in the indicated videos. (B) The yield points of uncured inks at two temperatures, 25 and −20 °C, for inks containing 65 vol % particles. Yield points are taken as ≥10% deviation from the linear viscoelastic region. Three samples were evaluated for each formulation, and the standard error of the mean is shown as the shaded region for each curve.

Figure 2A shows the viscosity response to decreasing temperature (down to −20 °C, the limit for our rheometer) for inks prepared from the two binder formulations and 65 vol % glass microsphere particles. While a consistent viscosity of approximately 2500 Pa·s is maintained from 40 to 0 °C, subzero temperatures induce a sharp increase in viscosity by 2 orders of magnitude for both binders. In addition, despite the higher amount of the diluent monomer HA in the 1:1 PEGDA:HA ratio, no viscosity reduction compared with the 2:1 PEGDA:HA ratio is observed. We verified the extrusion quality by attempting to print within the two distinctive regions, first at 25 °C and then at −30 °C. While extrusion proceeds continuously for a sample print at 25 °C shown in Video 1, at −30 °C, the extrusion quality deteriorated with frequent segments of no extrusion shown in Video 2, and the resulting print is incomplete. These results indicate that the selection of binder components based on low molecular weight and low glass transition temperature does not provide sufficient molecular mobility to reduce suspension viscosities at subzero temperatures. Since continuous extrusion, from the viscosity data, requires printing conditions above 0 °C, localized heating needs to be supplied to the ink if the environmental temperatures are in the subzero range.

While a high ink viscosity is detrimental to producing continuous extrusion, the observed high viscosities of both formulations at −30 °C can be beneficial to preventing slumping after extrusion. Since low-viscosity materials extruded in DIW are prone to structural deformation if the solidification step is not rapid enough,^14,45^ increasing shape retention of the extruded layers via a higher viscosity can be beneficial. However, based on the viscosity data and the large error in measurements made between −5 and −20 °C (due to the sensitivity of the equipment in measuring very rigid samples), as well as the failed extrusion for both binder formulations at −30 °C, it is difficult to verify if the ratio of PEGDA to HA plays a role in dictating the viscous behavior and shape retention capacities of inks at −20 °C. Yield point measurements can instead be conducted over a sweep of increasing stresses to pinpoint the stress value at which the ink experiences sufficient deformation to alter the suspension microstructure. An ink with a higher yield point post-extrusion is therefore favorable to maintain both the extruded shape and a homogeneous distribution of the ink components despite additional weight from subsequent layers.

The results in Figure 2B show high yield points obtained for 1:1 PEGDA:HA formulations at both ambient and −20 °C temperatures, with values of 31.4 ± 9.6 and 351.5 ± 46.6 Pa, respectively. Meanwhile, the behavior of the 2:1 PEGDA:HA formulation presents a different response to increasing stress at the two test temperatures with the noticeable presence of two yielding plateaus for this suspension at 25 °C. Only the initial plateau (describing the first deformation of the ink microstructure) is measured, and the yield point values of the 2:1 PEGDA:HA are recorded as 2.5 ± 0.3 Pa at 25 °C and 89.5 ± 18.8 Pa at −20 °C. The higher yield points in the 1:1 PEGDA:HA formulations are most likely due to steric stabilization provided by the alkyl chain on the HA molecule,^46,47^ which can contribute to shape retention by helping to resist particle settling after extrusion. In the 2:1 PEGDA:HA formulation, the initial plateau indicates the first instance of plastic deformation for this ink, but the presence of a secondary plateau, representing overall microstructure destruction, suggests that the reduced HA content provides a plasticizing effect rather than steric stabilization. For this formulation at 25**°**C, the low yield point is beneficial for limiting pressure demands on the extruder to initiate flow but also implies that very little force is required for this ink to deform once extruded. For both formulations, the higher yield points at low temperatures, consistent with the higher viscosities shown in Figure 2A, can further be explained by increased van der Waals interactions when the binder components are less mobile.^48^ At the low temperature, these yield points would present significant demands on the printing equipment and therefore reinforce the need for local heating to ensure extrusion. The results in Figure 2B suggest that while a higher HA content can provide greater structural stability of layers printed at 25 °C, steric stabilization is not necessary at −20 °C. By utilizing lower printing temperatures, inks can be formulated with reduced HA monomer content while retaining their shape retention capacities until adequate solidification occurs, thereby enhancing the quality of the resulting prints.

### 2.2 Curability

While the effect of temperature on the extrudability of the inks was significant enough to require localized heating to ensure extrusion, the curability of the inks at the low thermal extremes was also anticipated to differ from ambient conditions. From our observed rheological results, utilizing the extrudability criteria to narrow down the selection of monomers is not necessary since formulations must anyway remain heated to be extrudable. Instead, we turn to assessing the curability criterion to better determine if binder material selection is restricted for UV-assisted material extrusion at subzero temperatures. The curability criterion is a requirement for the printing process to provide sufficient solidification of layers for sequential layer extrusion. One challenge with curability stems from impaired light transmission through the extruded ink due to the presence of a large amount of particles, which can limit the height of the target printed layers and in turn limit the range of applications of material extrusion. It is therefore imperative to verify that a certain depth of cure can be achieved for the systems studied here and to investigate if subzero temperatures further limit the maximum achievable layer height. The ISO 4049 method, traditionally used to assess the curing of dental fillings that also have high solid loadings, translates to a valuable tool for rapidly assessing ink compositions for sufficient depths of cure before attempting to print at a range of temperatures.^49^ To tie this method to the printing process, the measured cure depth should ideally be greater than the desired layer height to maintain the shape of the layers and prevent sagging under gravitational forces and the weight of subsequent layers. In the case of the Lunar environment, which also features reduced gravitational forces, the solidification of inks can be even more essential in order to maximize shape retention. Some applications of DIW in microgravity demonstrated the heightened effect of surface tension in the absence of a gravitational force driving the extrudate downward, which resulted in print failures.^27^ While this effect can be mitigated, as was demonstrated with proper spacing between the extruder nozzle and the print bed,^26^ it is still crucial to ensure both rapid and sufficient solidification after deposition and good adhesion of the print to the print bed.

**Figure 3:**
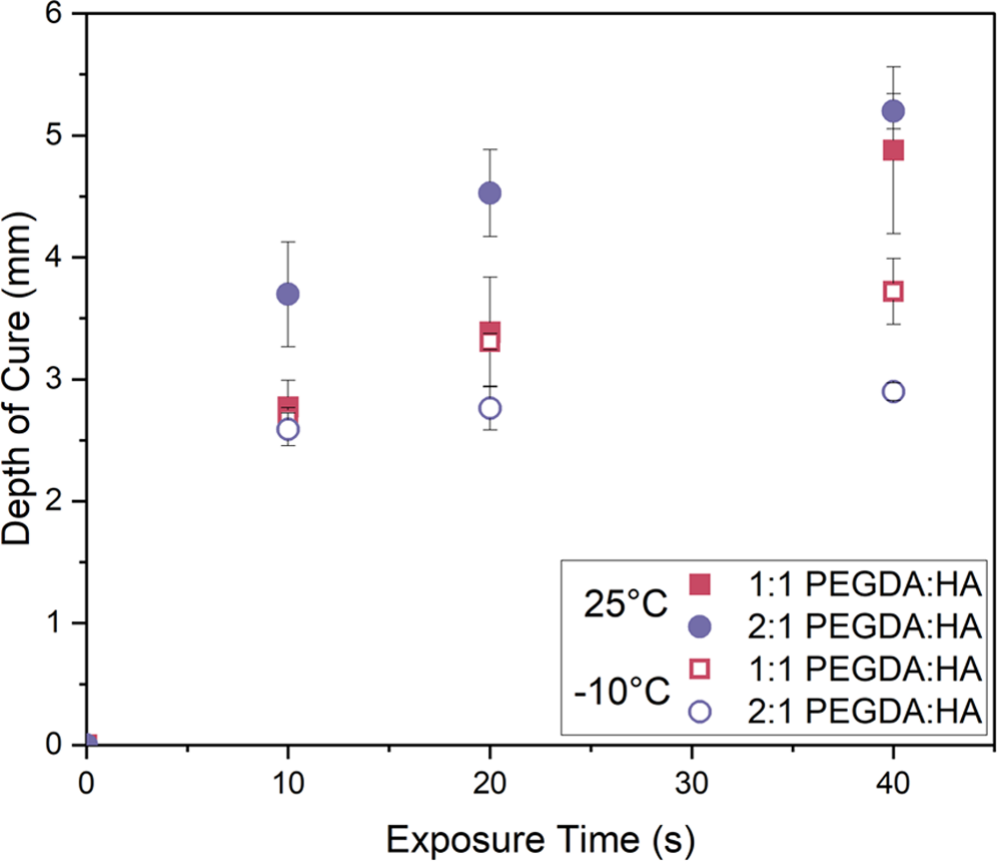
Cure depth measured by the ISO 4049 method for 65 vol % particle inks cured on a benchtop (25 °C) and inside a freezer (−10 °C) with varying exposure times to 5 W/m^2^ intensity UV light. As per the ISO 4049 method, recorded lengths of cure are halved before reporting, as shown here. The standard error is reported as the repeat of 3 samples.

The depth of cure of each formulation containing the different binders was therefore measured after UV curing had occurred at 25 ± 2**°**C on the lab benchtop and inside a laboratory freezer, which allowed for temperatures down to −10 ± 2 °C. The results in Figure 3 show that for these formulations, even short exposure times such as 10 s result in cure depths above the target layer height of 1 mm at both temperatures. After 20 to 40 s of UV exposure, most formulations do not experience a rise in cure depth as strong as that at shorter exposure times, and the cure depth begins to plateau. In ambient conditions, the 2:1 PEGDA:HA formulation, containing a greater concentration of PEGDA chains and more reactive end groups available on the polymeric molecule than the 1:1 PEGDA:HA, produces larger cure depths, but this is reversed at −10 °C, with the 1:1 PEGDA:HA formulation resulting in larger depths at all exposure times. We expect that the polymerization kinetics are slower for both binder formulations at −10 °C due to a reduction in molecular mobility, as expressed by the sharp viscosity and yield point increase in Figure 2A,B.

**Figure 4:**
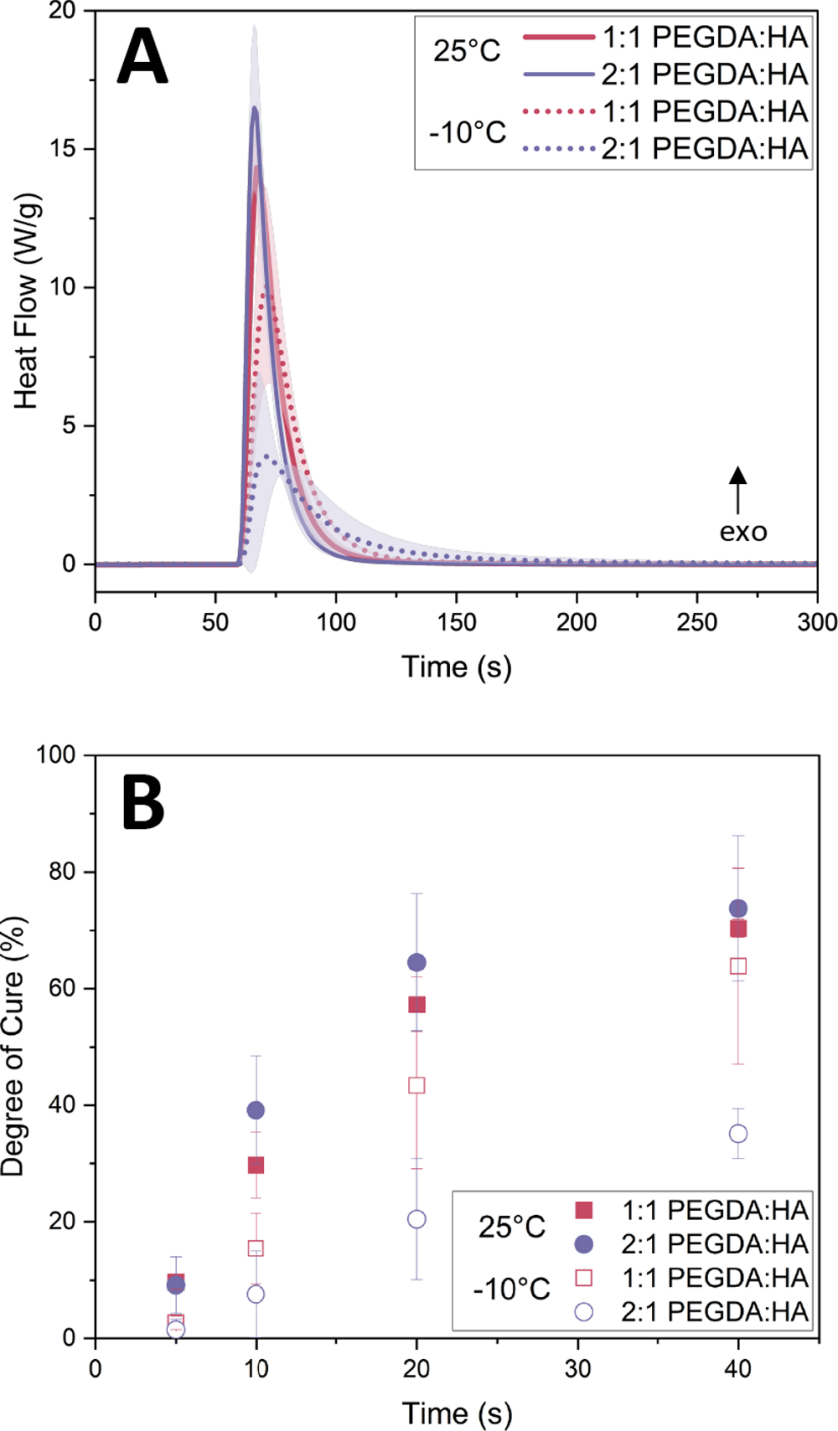
(A) Photo-DSC curves for the polymerization reaction. UV exposure begins at 60 s, and the lamp is turned off 5 min (300 s) later. The heat flow is normalized to the percentage of binder for each reported curve, as particles do not contribute to the polymerization reaction. The standard error across 3 samples is represented by the shaded region. (B) Calculated degree of cure from eq 1 and DSC curves reported above at two curing temperatures and along various time points during the active UV exposure. Standard error was reported across the mean of 3 samples.

During the printing process, maintaining good interlayer adhesion is important to prevent layer delamination and print failures. For UV-curing solidification, a slight degree of uncured binder can help promote the adhesion of the sequential layers. While the ISO 4049 method is a good guide for matching inks to specific print layer heights, it does not provide sufficient information on the polymerization of the binder to quantify the amount of unreacted monomers for the curability criterion. To understand how the printing temperature impacts the photopolymerization, it is necessary to investigate how the degree of cure with respect to time changes for different binder compositions at each temperature. To do so, we use the precise technique of photodifferential scanning calorimetry (photo-DSC), with results shown in Figure 4A,B. Although the capabilities of this equipment could allow characterization of the curing at our printing temperature of −30 °C, we carried out the photo-DSC analysis at −10 °C to establish a comparison with the results from the ISO 4049 test. The enthalpy of cure Δ*H*_*t*_, at various times, along with the heat flow *Q*_P_ and time *t*_P_ recorded at the exothermic peak, is extracted from the photo-DSC curves in Figure 4A and is reported in Table 1. These were used to compute the extent of polymerization, the maximum heat of the reaction, and the time to the highest conversion of vinyl bonds.^50^ The degree of cure was calculated by running the same procedure on a binder sample containing no particles and measuring Δ*H*_binder_, the maximum enthalpy of cure in the absence of light scattering, absorption, and reflection from the particles. The degree of cure at various times *t* is plotted in Figure 4B and was computed from the formula in eq 1(38,50,51)
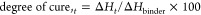
1

**Table 1:** Reported Values of *Q*_P_, Δ*H*_*t*_, and *t*_P_ for Inks Cured at Two Different Temperatures. Standard Error Reported for the Mean of 3 Samples

	peak height (W/g), *Q*_P_		enthalpy of cure (J/g), Δ*H*_*t*_		time to peak (s), *t*_P_	
	–10 °C	25 °C	–10 °C	25 °C	–10 °C	25 °C
1:1 PEGDA:HA	10.1 ± 3.6	14.6 ± 2.0	214.0 ± 50.9	222.6 ± 3.3	11.3 ± 0.3	7.7 ± 0.7
2:1 PEGDA:HA	4.8 ± 2.4	16.5 ± 3.0	133.0 ± 21.7	207.8 ± 16.5	15.0 ± 3.5	6.3 ± 0.3

At ambient conditions, both formulations exhibit similar polymerization kinetics. At −10 °C, we identified a reduction in cure kinetics, visible in the observed broader and shorter peaks for the 2:1 PEGDA:HA formulation and the correspondingly smaller Δ*H*_*t*_ and *Q*_P_ and the longer *t*_P_. In addition, in Figure 4B, the 2:1 PEGDA:HA formulation cured at −10 °C shows the lowest degree of cure at only 35 ± 4% after 40 s of UV exposure. For the 1:1 PEGDA:HA formulation, the difference in the enthalpy of cure and the heat flow between 25 and −10 °C are not statistically significant (on the *t*-test basis), but there is a delay to the onset polymerization at −10 °C, as the highest conversion of the vinyl bonds happens a few seconds later than at 25 °C. Also, in the 1:1 PEGDA:HA formulation, the degrees of cure measured at 25 and −10 °C shown in Figure 4B initially show statistical differences (run through a *t*-test) at the short UV exposure time scales, but these differences become statistically insignificant after 20 s. The data presented in Figures 4A–B indicate that both ink formulations contain residual uncured monomers at 25 and −10 °C, with a greater concentration of unconverted monomer present when curing at the lower temperature. This indicates that printed layers at −10 °C could maintain better intralayer adhesion. However, the low cure degree of the 2:1 PEGDA:HA formulation could lead to layers extruded from this ink having a lower structural strength and could potentially deform more easily.

Overall, the trend seen in the depths of cure plotted in Figure 3 matches those for the degrees of cure in Figure 4B for both ink formulations at 25 and −10 °C. This suggests that the reduction in layer cure depth likely stems from subzero temperatures slowing down the polymerization kinetics in UV curing. At room temperature, a greater extent of polymerization occurs when more acrylate functional groups are present, as we have demonstrated here with a higher concentration of PEGDA chains. Thus, increasing the functionality of the selected monomer is a viable way to optimize the cure depth of formulations in ambient conditions. It could be possible to optimize cure depth at low temperatures by using a dual cure binder and harnessing the heat from UV polymerization to trigger a thermal-curing mechanism. In this manner, the low-temperature environment could also provide the added benefit of preventing premature reactions in the thermal cure mechanism. For binders that rely solely on UV curing, additional functional groups could help in achieving greater reactivity, but these additional groups can also increase the molecular weight of the monomers and reduce the molecular mobility, which could translate to a decrease in cure depths and cure degrees at cold temperatures.

In addition to reducing molecular mobility of the monomers at the colder temperatures, the poly(ethylene glycol) repeating units in PEGDA are known to form soft crystalline phases below 10 °C, which could contribute to a reduced extent of polymerization and depth of cure^52^ (see crystalline phases identified in Figures S2 and S3). As we show in Figure S2A, for the uncured inks, endothermic crystal melting peaks are visible around −20 to 35 °C. However, after curing at −10 °C, the crystal melting peaks disappear for the 1:1 and 2:1 PEGDA ratios and are also not present in the XRD spectra in Figure S3 (for pure PEGDA, the peak is still present). We expect that the crystalline to amorphous phase change is due to fast reaction kinetics, promoted by HA’s small molecule size and the heat released during vinyl bond conversion. This indicates that the monomer HA is beneficial in enabling PEGDA to take on an amorphous state during photopolymerization. Therefore, for printing applications where the binder formulation requires monomers or prepolymers with the potential to crystallize at subzero temperatures, a binder mixture with small and highly reactive monomers should be considered.

### 2.3 Cross-Linked Microstructures

**Figure 5:**
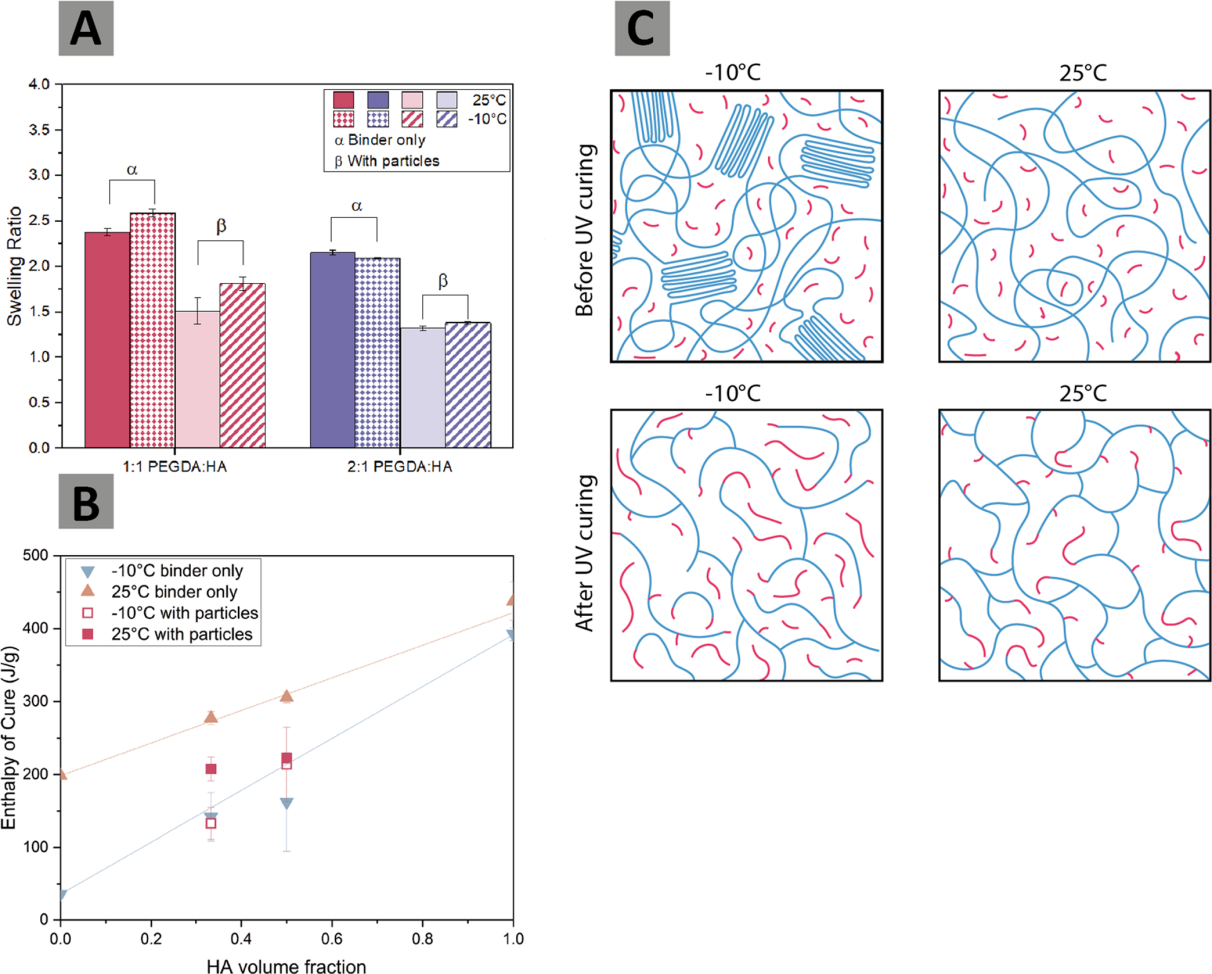
(A) Swelling ratios (normalized to binder weight in each sample) for both binder-only (α) and particle-containing (β) samples cured at 25 °C (solid fill) and −10 °C (patterned fill). The samples were swollen in chloroform, and the wet mass after the removal of excess solvent was recorded. The representative mean and standard error are reported here for the three samples. (B) Monomer contributions to the heat of curing as reported through photo-DSC. The 1:1 and 2:1 PEGDA:HA formulations are indicated at 0.5 and 0.33 HA volume fractions, respectively. Triangle symbols do not contain particles, but square symbols do. Extrapolation of a linear fit for the binder-only data at both 25 and −10 °C is also shown. Each data point is the average of three samples with the corresponding standard error of the mean reported. (C) Illustration of the proposed microstructure before and after curing of the 1:1 PEGDA:HA formulation. PEGDA chains are represented as long blue chains, and HA monomers are represented as short red lines. At −10 °C before curing, PEGDA forms crystalline phases. The heat produced by polymerization is sufficient to melt these crystalline phases. However, a different microstructure is formed at −10 °C after curing, with more short copolymer chains created, rather than the tightly cross-linked network that forms at 25 °C. This causes a reduction in cross-linking density for the 1:1 PEGDA:HA formulation cured at −10 °C.

Although the curability of photopolymer binders can be achieved despite PEGDA crystallization at low temperatures, at the onset of polymerization and the early stages of crystalline phase melting, the ratio of PEGDA to HA monomer can result in different cross-linked microstructures. We therefore considered the type of microstructure that forms out of the two formulations when printed at both 25 and −10 °C through a joint analysis of the cross-linking density and heat of reaction for PEGDA versus HA bonds. First, we performed swelling experiments, shown in Figure 5A, to assess changes in the cross-linking density of different PEGDA to HA ratios since the degree of swelling is inversely related to the cross-linking density.^53^ Second, we looked at the heat produced for bonds forming between PEGDA molecules and between HA molecules and assessed how the curing temperature affects the likelihood of these bonds forming both for pure monomers and for our 1:1 and 2:1 mixtures (Figure 5B). This allows us to complement our observations of the cross-linking density from the swelling experiments and to generate explanations based on possible microstructures before and after UV curing at both −10 and 25 °C (Figure 5C).

In Figure 5A, we assess the changes in cross-linking density due to curing temperature. Here, our definition of the cross-linked state is restricted solely to the rigid network formed from long and interconnecting PEGDA-based chains. Short copolymers, made of a PEGDA chain terminated at both reactive ends by HA monomers with a single reactive acrylate group, and linear HA-based short chains are not considered as part of the cross-linked network since they are not covalently bonded to the larger web. In addition, due to their small size, the HA monomers and small chains can be separated from the cross-linked network with the appropriate solvent. We identified chloroform as a good solvent to solubilize these small fragments not attached to the larger cross-linked network, and we measured the swelling ratio of both particle-containing and binder-only samples cured at −10 °C and at 25 °C. The resulting swelling ratios, shown in Figure 5A, are calculated as described in eq 2, where *m*_*wet*_ is the mass of the swollen, wet sample after removal of the excess solvent, and *m*_*i*_ is the initial dry mass of the sample.
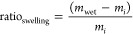
2

Across the 2:1 PEGDA:HA mixtures, samples containing particles have significantly reduced swelling ratios compared to binder-only samples, both at 25 and −10 °C. However, the cure temperature had no statistically significant effect (on the *t*-test basis) on the swelling ratios of the 2:1 PEGDA:HA formulation when comparing the binder-only samples individually from the particle-containing samples and vice versa. Since the rigidity of the particle network limits the ability of the binder to swell, a decrease in the swelling ratio of the samples with particles is expected when compared to the binder-only samples. The lack of difference in swelling ratio across the two temperatures, for both the binder-only and particle-containing samples individually, also suggests that the cross-linking density formed for the 2:1 PEGDA:HA ratio remains similar. Despite the higher PEGDA ratio causing this formulation to be less mobile and more likely to crystallize at cold temperatures, the small amount of HA monomer is sufficient to produce a cross-linking microstructure similar to the one created when curing at 25 °C. The 2:1 PEGDA:HA formulation therefore appears to be a good printing ink recommendation for printing across a wide range of temperatures.

For the 1:1 PEGDA:HA samples, the temperature had a more significant impact on the swelling ratios. First, we observed overall higher swelling ratios in the 1:1 PEGDA:HA samples than in the 2:1 PEGDA:HA. Second, for the 1:1 PEGDA:HA formulations at −10 °C, the swelling ratios are greater than those at 25 °C. This observation applies to both the binder-only and particle-containing samples. The higher swelling ratio in the 1:1 PEGDA:HA samples compared to the 2:1 PEGDA:HA samples can be explained by the lower concentration of PEGDA available to form the cross-linked network, and so a lower cross-linking density is created. However, when focusing solely on the 1:1 PEGDA:HA and comparing the samples prepared at the two temperatures, we can also deduce that curing at −10 °C results in an even lower cross-linking density. We hypothesize that the lowest cross-linking density measured for the 1:1 PEGDA:HA mixture at −10 °C is due to more short-chain copolymers and linear HA chains forming. To verify our hypothesis, we measured the mass loss in the swollen samples after drying them (see Figure S4). As detailed in the Supporting Information, the higher mass loss in the 1:1 PEGDA:HA sample cured at −10 °C is likely caused by the removal of short-chain copolymers and linear HA chains during the solvent extraction step. This indicates that a higher concentration of the small and highly reactive HA monomer results in a microstructure change when cured at −10 °C compared to 25 °C. Therefore, an equal volumetric ratio of PEGDA and HA monomers, although exhibiting faster cure kinetics than the 2:1 ratio, is not a suitable choice for maintaining a consistent cross-linking density across curing temperatures.

To further understand how these short-chain copolymers could form, we also analyzed the enthalpy associated with PEGDA–PEGDA, HA–HA, and PEGDA–HA bond formation. Figure 5B shows the total enthalpy of cure as a function of the volume fraction of the HA monomer in the binder mixture when the curing temperature changes between 25 and −10 °C. Since the conversion of the vinyl bond in the reactive acrylate group is exothermic, a higher enthalpy of cure is expected to correlate with more bonding. We can isolate the effect of temperature on the formation of PEGDA–PEGDA and HA–HA bonds by looking at the pure PEGDA binder-only (HA volume fraction = 0) and the pure HA binder-only (HA volume fraction = 1), respectively. A comparison between the enthalpies of cure reveals that bond conversion is always higher between HA monomers than between PEGDA chain ends at both 25 and −10 °C. At −10 °C, however, this difference in enthalpy of cure is more pronounced than at 25 °C, with HA–HA bonds resulting in 392.5 ± 9.0 J/g versus only 36.1 ± 2.6 J/g for the PEGDA–PEGDA bonds. This indicates that HA monomers polymerize more readily than PEGDA chains, likely due to their smaller size and higher molecular mobility. The cold temperature enhances this aspect more intensely due to the added effect of PEGDA crystalline phases when curing occurs at −10 °C.

For the 1:1 and 2:1 PEGDA:HA mixtures of monomers, the enthalpy of the curing reaction is the sum of bonds forming between HA–HA, PEGDA–HA, and PEGDA–PEGDA. The respective enthalpy from each type of bond formation is affected by both monomer mobility and PEGDA crystallization. For the 2:1 PEGDA:HA (0.33 vol fraction HA) and 1:1 PEGDA:HA (0.5 vol fraction HA) at 25 °C, the enthalpy of cure appears to depend linearly on the volume fraction of the HA monomer. However, the slope of the line of enthalpy of cure vs HA volume fraction is higher when the curing temperature is −10 °C. As a result of this data, and since we know the molecular mobility of PEGDA decreases significantly at the cold temperature due to crystallization, we hypothesize that the increase in the slope of the line at −10 °C could stem from polymerization being initiated more prevalently by the HA monomers. Furthermore, since HA-based bonds can only form linear chains because HA has only one acrylate group, this would support our cross-linking density conclusions drawn from Figure 5A. We present a visual interpretation, drawn from our combined cross-linking density and heat of curing results, of our expected binder microstructure for the 1:1 PEGDA:HA mixture before and after UV curing at both 25 and −10 °C in Figure 5C. As we show small chain copolymers forming to a greater extent at −10 °C, we also highlight that the 1:1 ratio of PEGDA to HA monomer does not result in reproducible cross-linked microstructures across a wide temperature range. This changing microstructure due to temperature for the 1:1 PEGDA:HA mixture suggests that there is a trade-off between fast reactivity and consistent cross-linking density for curing at −10 °C. These baseline results with pure HA and pure PEGDA systems can be used to assess the structures formed by the mixtures with and without particles.

So far, the enthalpy of cure analysis was done on the binder only, but it is also important to examine the suspensions containing particles. Still, in Figure 5B, a comparison between the particle-containing and the binder-only samples shows reduced enthalpies of cure at 25 °C for the particle-containing inks but greater or similar enthalpy values to the binder-only samples at −10 °C. Note that the enthalpy of cure is normalized to the amount of binder, so we would expect the enthalpy of cure to be similar to binder-only samples for both temperatures if the particles do not play a role beyond taking up space. However, the enthalpy of cure can decrease with particles present due to light interaction with the particles, radical diffusion limitations, and heat conduction to the particles.^54^ Since the enthalpy of cure is approximately the same with and without particles at −10 °C and we know that the particles do reflect, absorb, and scatter light, this suggests that the high content of the glass microspheres in the particle-containing inks may provide a small extent of thermal shielding.^55^ This may help to maintain the suspension temperature above −10 °C around the particles, which would promote faster cure kinetics.

Similarly to our analysis of the slope change at −10 °C for the binder-only samples, in the particle-containing mixtures, the enthalpy of cure for the 1:1 PEGDA:HA at −10 °C is noticeably higher than the enthalpy of cure for 2:1 PEGDA:HA. This suggests that despite the reduced free volume from the inclusion of the particles, both higher reactivity of the HA monomer and PEGDA crystallization still result in a microstructure change. This is in agreement with our swelling ratio results shown in Figure 5A. As we illustrate in Figure 5C, we expect a greater extent of linear small chains and a lower overall cross-linking density. In the scope of UV cure additive manufacturing, the depth of cure and degree of cure measurements are typically considered important measurements, but these results indicate that the microstructure formed during photopolymerization can also vary. Here, we have shown two important and interrelated factors: the molecular mobility of monomer species at low temperatures and the formation of small linear HA chains and copolymers. These become important to characterize prior to printing at low temperatures in order to assess if the homogeneity of the ink microstructure could deviate from what would be expected at ambient temperatures.

**Figure 6:**
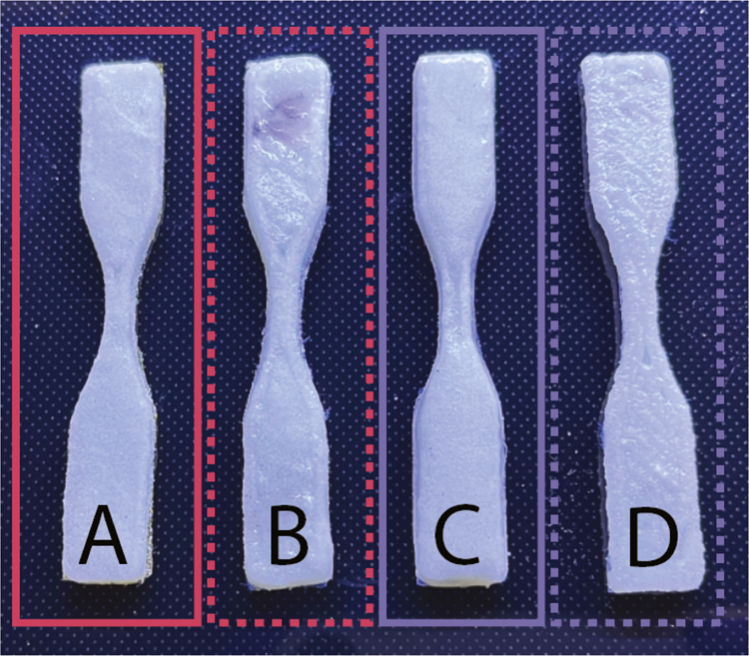
One representative sample of the printing quality for each of the different formulations at the two printing temperatures. From left to right: (A) 1:1 PEGDA:HA at 25 °C, (B) 1:1 PEGDA:HA at −30 °C, (C) 2:1 PEGDA:HA at 25 °C, (D) 2:1 PEGDA:HA at −30 °C. 1:1 PEGDA:HA inks are surrounded in red and 2:1 PEGDA:HA inks are surrounded in purple. Solid outlines denote samples printed at 25 °C, and dashed outlines denote samples printed at −30 °C.

Despite the differences in cure depth and cure degree and the change in the microstructure at subzero temperatures, all suspensions were printable at −30 °C, the target temperature of our printing environment test apparatus. For the 25 °C prints, both the nozzle and bed were at 25 °C, while for the −30 °C prints, the nozzle was kept at 23 ± 3 °C, and the bed and environment were at −30 °C. The UV exposure was continuous during the extrusion moves, with a printing speed of 10 mm/s and an intensity of 5 W/m^2^ (matching the UV exposure intensity for the photo-DSC and ISO 4049 experiments). After printing, we performed a 1 min postcuring step at a higher UV intensity in a dedicated curing box (the degree of cure after this step for each formulation is shown in Figure S5A,B). Example prints for the two formulations printed at both 25 °C and −30 °C are shown in Figure 6. Despite low cure degrees for the 2:1 PEGDA:HA formulation at −10 °C, the high viscosity/yield point at subzero temperatures allows the printed ink to withstand the weight of additional layers. There is little visible difference in print quality between the formulations and for the different temperatures, indicating similar extrusion behavior and the ability to print subsequent layers. However, the changes in the depth and degree of cure at the low temperature, as shown in Figures 3 and 4B, combined with the microstructure change, could still result in different mechanical performances.

### 2.4 Tensile Performance

**Figure 7:**
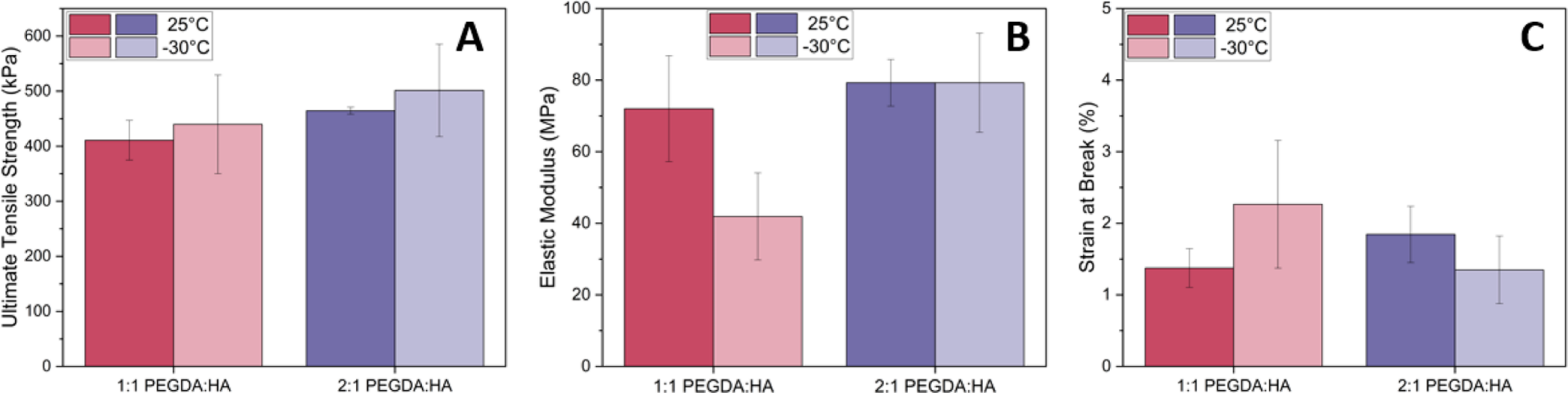
Tensile properties for ASTM D6378 type V prints containing 65 vol % particles and produced at both 25 and −30 °C in an enclosed environment: (A) ultimate tensile strength, (B) elastic tensile modulus, and (C) strain at break. Five samples were tested for each formulation at both temperatures, and the standard error of the mean of the five samples is presented.

For composite systems, the mechanical performance is driven by both the solid filler and the surrounding binder. As we had noted a lower cross-linking density and a likelihood for more small copolymers and linear HA chains when the 1:1 PEGDA:HA formulation was printed at −10 °C, we also needed to verify if these caused noticeable differences across the mechanical properties. The dogbones shown in Figure 6 were therefore tested in tension to assess their ultimate tensile strength, elastic tensile modulus, and strain at break. In Figure 7A–C, we present the tensile results obtained from the dogbones printed at both 25 and −30 °C. All dogbones were cured for the same amount of time (approximately 40 s per layer), regardless of temperature, and all dogbones received the same 1 min postcure treatment at the completion of the print. First, the ultimate tensile strength in Figure 7A remains statistically (*t*-test) similar across both 1:1 and 2:1 PEGDA:HA inks printed at both 25 and −30 °C. Second, the elastic tensile modulus in Figure 7B presents statistically significant differences (on the basis of the *t-*test) across samples and printing conditions. Starting with the 1:1 PEGDA:HA formulation, the elastic modulus is 72.0 ± 14.8 MPa for prints produced at 25 °C and 41.9 ± 12.2 MPa for prints produced at −30 °C. For the 2:1 PEGDA:HA formulation, printing temperature does not appear to result in significant changes to the elastic modulus of the warm and cold printed samples. However, when comparing the 1:1 PEGDA:HA and 2:1 PEGDA:HA at −30 °C, there is a significant difference in the elastic modulus, where the values are 41.9 ± 12.2 and 79.3 ± 13.9 MPa, respectively. We do not see a statistically significant difference (*t-*test) in the elastic modulus for the 1:1 and 2:1 PEGDA:HA formulations when they are printed at 25 °C. Finally, no statistically significant difference (*t*-test) was measured for any comparisons of the mean strain at break in Figure 7C for both formulations and at both temperatures.

The similar results for the ultimate tensile strength and strain at break across formulations and across temperatures (Figure 7A,7C) suggest that at this high content of solid particles (65 vol %), both the tensile strength and elongation may be primarily determined by the solid particle phase and frictional contacts between particles rather than by the surrounding binder. In contrast to the ultimate tensile strength and strain at break, which are measured after considerable stress is applied, the elastic modulus tracks the deformation response in the early stages of the applied stress. As such, because our particles are relatively larger and stiffer than our binder molecules, most of the early stresses in the ink are dissipated through the binder first. Therefore, the changes in the cross-linking microstructure, demonstrated in Figure 5A–C, are more clearly reflected in the elastic modulus.

For the 2:1 PEGDA:HA samples printed at 25 and −30 °C, the lack of differences across the elastic modulus values is consistent with our observation of the swelling ratios for this formulation and indicates that the cross-linking density also remains similar. Likewise, a comparison of the elastic modulus between the 1:1 and 2:1 PEGDA:HA prints at 25 °C reveals no differences, which also suggests similar stiffness despite different HA contents. However, at −30 °C, the measured elastic moduli indicate that the 1:1 PEGDA:HA formulation has a lower stiffness than the 2:1 PEGDA:HA, which hints at a microstructure change. For the 1:1 PEGDA:HA formulation, the lower elastic modulus at −30 °C compared to 25 °C agrees with a lower cross-linking density that was concluded from the higher swelling ratio in Figure 5A. The lower elastic modulus also supports our proposed microstructures shown in Figure 5C since small copolymers and linear HA chains that are not covalently attached to the larger cross-linked network would not contribute to the stiffness of the cured binder. Since the formation of these species is not localized, stiffness heterogeneities can form across the print. This can increase the risk of premature and uncontrolled failure of the printed part. Therefore, the 2:1 ratio of a longer chain prepolymer (PEGDA) to a small monomer (HA) is a more promising candidate for further DIW efforts at subzero temperatures since the mechanical properties are maintained despite slower curing kinetics and PEGDA crystallization. Future work will seek to determine whether this ratio holds true with different monomers.

Throughout this analysis of the mechanical properties across both of our inks printed at 25 and −30 °C, we have shown how different ink formulations do not produce the same print quality when we translate the ambient printing process to subzero temperatures. To achieve a specific ultimate tensile strength or elongation at −30 °C, the selection of binder materials can be driven by the formulation design parameter of extrudability in the presence of a high content of solid particles and ensuring sufficient solidification post-extrusion. But for a target elastic modulus, the binder formulation should be designed with curability in mind and monomers need to be selected to promote the formation of a cross-linked network rather than linear chains and small copolymers at subzero temperatures.

## 3 Conclusions

The formulation design of DIW inks with high solid suspensions to be printed in challenging environments, such as extreme cold, requires an evaluation of the performance of the different ink components under those unique conditions. In this study, we targeted two formulation design guidelines, extrudability and curability, to develop inks composed of 65 vol % solid particles suspended in a photopolymerizable binder of HA and PEGDA monomers. We probed the inks’ performance with respect to curability and extrudability at both ambient and subzero temperatures. We showed that for the extrudability criteria, inks must remain heated and that curability requirements to ensure sufficient solidification after extrusion can be met with cure depth and cure degree assessments. In addition to the two criteria, we demonstrated that the ratio of PEGDA to HA monomers can produce different cross-linked microstructures when cured at subzero temperatures.

For a ratio of 1:1 PEGDA:HA, we demonstrated the formation of a greater amount of small copolymers and linear HA chains when printing at subzero temperatures. This resulted in a weak structural integrity of the printed parts. We showed this with a lower cross-linking density and a decrease in elastic tensile modulus when compared to this same ink printed at 25 °C. This change in microstructure can be mitigated by increasing the concentration of PEGDA to a 2:1 PEGDA:HA ratio. Despite showing slower cure kinetics, this formulation maintains a consistent cross-linking density across the temperature range and retains its tensile properties from 25 to −30 °C. While we do not account for how binder–particle interactions could also influence the curing kinetics at low temperatures, we do demonstrate that the selection of the ratio of monomers for curing at low temperatures is a trade-off between fast reactivity and a consistent cross-linking density. We expect that with the increasing momentum in space exploration, this study will contribute to new ink designs for additive manufacturing that facilitate the translation from ambient conditions to new and challenging environments.

## 4 Materials and Methods

### 4.1 Ink Formulation

To generalize the applicability of the formulation design, a model particle system was used. Solid glass microspheres (Potters Industries) in two size ranges were selected to produce a bimodal ratio of particles and allow an increase in the overall solid loading without nearing the jamming transition. The total solid loading was maintained at 65 vol %, with larger particles (A-1820, D_avg_ = 270 μm) comprising 65% and smaller particles (A-3000, D_avg_ = 35 μm) comprising 35% of the total solid volume. The photocurable binder used in this study was sourced from Sigma-Aldrich and is a mixture of poly(ethylene glycol) diacrylate (PEGDA 700), a difunctional acrylate prepolymer, and hexyl acrylate (HA), a monofunctional monomer. These were chosen for their relatively low glass transition temperatures (T_g_) in both the uncured state (T_g_ = −57 °C for HA and T_g_ = −39 °C for PEGDA) and as a final cross-linked network (averaging around −43 °C for both 1:1 and 2:1 PEGDA:HA), to optimize processing at subzero temperatures. The ratio of PEGDA to HA was varied in order to create different cross-linking densities upon curing and also to assess how binder composition may affect extrusion and curing kinetics in subzero temperatures. Due to extensive light interaction from such a high loading of filler material, the photoinitiator phenylbis (2,4,6-trimethylbenzoyl)-phosphine oxide (BAPO, Sigma-Aldrich) was selected. BAPO is commercially recognized as optimal for use in dense systems to optimize cure depth at a concentration of 0.5 wt %.

Twelve milliliters ink batches were produced by combining the materials in ratios listed in TABLE 2. Binder resins containing PEGDA and HA were premixed in either 1:1 or 2:1 vol/vol ratios. The resin was added to the particles, and the BAPO photoinitiator was incorporated to comprise 0.5 wt % of the binder weight. Inks were thoroughly mixed in a Flacktek 400.2VAC-L dual axis centrifugal (DAC) mixer for 1 min at 1000 rpm, followed by 45 s at 0 rpm, and last for 45 s at 1500 rpm.

**Table 2:** Ink Compositions for the Two Formulations

formulation	small particles (g)	large particles (g)	PEGDA (mL)	HA (mL)	BAPO (g)
1:1 PEGDA:HA	6.83	12.68	2.1	2.1	0.0217
2:1 PEGDA:HA	6.83	12.68	2.8	1.4	0.0217

### 4.2 Rheological Characterization

Rheological measurements were performed on a TA Instrument DHR-3 rheometer equipped with 20 mm cross-hatched parallel plates. The gap distance was set to 1000 μm, and a thermal cover was utilized to minimize the exposure of the ink to ambient lighting and to prevent thermal losses. A predetermined preshear procedure was applied to homogenize the samples and remove stresses associated with loading the samples on the plates.^44^ Two experiments were conducted in the oscillatory mode, starting with amplitude stress sweeps from 0.001 to 10,000 Pa at a frequency of 10 rad/s to assess the linear viscoelastic region of the inks and identify their yield point. The amplitude stress sweeps were performed at both 25 and −20 °C (the lowest achievable temperature with a rheometer chiller), and the yield point was determined as the stress value at which the storage modulus decreased by 10% or more from the average value recorded in the linear viscoelastic region. Temperature sweeps were then conducted, also in the oscillatory mode and within the linear viscoelastic region of the inks, to minimize microstructure deformation. Sweeps were conducted from 40 to −20 °C at 1 Pa (defined as being within the linear viscoelastic regions of all samples).

### 4.3 Depth of Cure and Photo-DSC

The maximum achievable cure depth was investigated according to the ISO 4049 procedure. A flexible mold with an inner diameter of 1/8 in. was filled with ink and exposed to a UV light source with set intensity at 5 W/m^2^ and exposure times ranging from 5 to 40 s. This method was performed both on a benchtop at room temperature and in a freezer set to −10 °C ± 2 °C. The cured parts were then removed from the mold and placed in a bath of isopropanol to wash out any uncured sections. Dried parts were imaged, and the half-length was measured through the ImageJ software.

Curing kinetics were investigated by means of photo-differential scanning calorimetry (photo-DSC). A Mettler Toledo DSC 3+ equipped with an Omnicure 2000 UV Lamp filtered at 365 nm was used. The relative intensity of the lamp was adjusted to 5 W/m^2^. Approximately 40–60 mg of uncured samples were loaded in 40 μL aluminum crucibles so that the top of the ink was level with the rim of the crucible. Curing experiments were conducted isothermally at 25 and −10 °C. An initial 1 min equilibration step without UV exposure was followed by 5 min of continuous UV exposure, after which calorimetric data continued to be recorded for an additional 2 min in the dark. This procedure was repeated twice more to deconvolute heating effects due to the polymerization reaction from local heating due to UV irradiation.

The presence of the crystalline phases was demonstrated by running several heating and cooling cycles on the uncured and cured formulations. The samples were heated from 25 to 100 °C at 20 °C/min, held at 100 °C for 2 min, then cooled to −30 °C at 20 °C/min, held at −30 °C for 5 min, and heated again to 100 °C at 20 °C/min. The heating and cooling steps between −30 and 100 °C were repeated once more, and the last heating cycle from −30 to 100 °C was utilized to extract the data plotted in the Supporting Information.

### 4.4 UV-Assisted Robocasting

Printing was conducted on a Hyrel Engine Standard Resolution printer equipped with a Volcano 25 print head and fitted with a 365 nm UV array with a tunable intensity positioned around the nozzle. Immediately after mixing, the inks were loaded into a stainless-steel syringe cartridge. A 14G (ID = 1.6 mm) black UV-blocking nozzle was attached. Due to placing the syringe in an upright arrangement and loading the ink from the tip-end of the syringe barrel, no degassing step was performed, as trapped air bubbles were allowed to escape when the ink was manually pushed to the nozzle tip. Although the recommended ratio of nozzle diameter:largest particle size is 10:1,^56^ and our ratio is ∼7.71:1 (from the d_50_ particle size values), this was the largest commercially available nozzle in the UV product line. Loaded syringes were used within 4 h for printing. We conducted preliminary extrusion tests to ensure that continuous extrusion could be achieved without nozzle clogging. The UV light intensity was set to emit 5 W/m^2^ to match the intensity used during the depth of cure and photo-DSC measurements and was activated during detected print head moves. The print speed was set to 10 mm/s, the piston-driven extrusion rate was set to 250 pulses/μL, and the layer height was specified as 1 mm. The printing substrate was a PET sheet for the ease of removal of the otherwise brittle samples.

Printing was performed at 25 °C on a standard benchtop. Printing at −30 °C was accomplished by enclosing the printer in a chamber with continuous gaseous nitrogen (GN_2_) flow and regulating the influx of cold GN_2_ via a solenoid controlled by a custom LabView program. For cold printing, the Volcano 25 head was loaded with ink and attached to the ESR printer at ambient temperature before sealing the chamber and initiating cooling. With this setup, printing occurs approximately 2 h after ink loading. Prior tests conducted to assess any demixing in ink showed that despite the delay, these inks could still extrude homogeneously. Due to the sensitivity of the binder to temperature and in order to maintain extrudability, the syringe barrel and nozzles were kept heated between 20 and 40 °C during printing operations at −30 °C. The large temperature range was due to extreme thermal gradients occurring near the nozzle exit, and it was necessary to raise the temperature in the syringe to ensure that the suspension would be extrudable at the nozzle tip. Following printing, the samples were moved to an Elegoo Mercury curing machine and postcured for 1 min. In the case of the samples produced at −30 °C, postcuring occurred 2 h after printing, as it was necessary to warm the chamber before retrieving the samples.

### 4.5 Tensile Testing

Tensile testing of the printed ASTM D638 type V dogbones was conducted on a TA Instruments DHR-3 rheometer equipped with a rectangular tension accessory. The dimensions of the gauge region of the samples were measured prior to testing and programmed into the TRIOS software. Samples were tightened in the top and bottom holders with a torque driver set to 50 μN/m. The axial force was zeroed before each test, and the pull rate was set to 5 mm/min. Five samples were tested for each formulation printed at either 25 or −30 °C.

### 4.6 Swelling Ratios

In order to evaluate the cross-linking density of the cure inks, samples were created from the same binder formulations with and without particles, and the samples were cured at either 25 or −10 °C in a small silicone mold. The mass of the cured gels was recorded, and they were left to each swell individually in 3 mL of chloroform for 48 h. The excess solvent was then carefully removed using pipettes, and the mass of the swollen gel was recorded. To obtain the dry mass, the swollen gels were then left to dry in a convection oven at 60 °C for 24 h.

### 4.7 X-ray Diffraction

The 1:1 and 2:1 PEGDA:HA formulations were poured into the same molds used for the swelling ratios and were cured on the benchtop at 25 ± 2 °C or in the freezer at −10 °C. UV exposure was set to an intensity of 5 W/m^2^ and lasted for 60 s. A Malvern PANalytical Empyrean X-ray diffractometer equipped with a copper anode and a PIXcel detector was then used to assess the presence of crystalline phases in cured binder mixtures. Scans were run at 2Θ angles between 10 and 80°. A divergence slit of 1/8°, a 4 mm mask, a 0.04 rad soller slit, and a 1° antiscatter slit were used.

## Supporting Information Available

Additional experimental characterization of the formulations, including shear rheology, crystalline phase assessment via DSC and XRD, mass loss recorded after the swelling experiments, confirmation of the need for the postcure step, and a computation of the number of acrylate functional groups (PDF)
Extrusion proceeds continuously for a sample print at −30 °C with localized heating (MP4)
Extrusion quality deteriorated with frequent segments of no extrusion at −30 °C and no localized heating (MP4)
