# Supplementary material for: Applicability of UV-Curable Binders in High Solid Suspensions for Direct-Ink-Write 3D Printing in Extremely Cold Temperatures
Source: ACS Appl Mater Interfaces. 2023 Oct 20;15(43):50378–90. doi: 10.1021/acsami.3c11742 (PMC10623509; doi:10.1021/acsami.3c11742)
Supplement: Supplementary file 1 — am3c11742_si_001.pdf [file am3c11742_si_001.pdf]

Supporting Information

Applicability of UV-Curable Binders in High Solid Suspensions for Direct Ink Write 3D Printing in Extremely Cold Temperatures

Alexandra Marnot[1], Lena Konzelman[2], Jennifer M. Jones[3], Curtis Hill[4], and Blair Brettmann[1,5*]

[1]School of Chemical and Biomolecular Engineering, Georgia Institute of Technology, Atlanta, GA 30332, USA
[2]George W. Woodruff School of Mechanical Engineering, Georgia Institute of Technology, Atlanta, GA 30332, USA
[3]NASA Marshall Space Flight Center, Huntsville, AL 35898, USA
[4]NASA Marshall Space Flight Center, Jacobs Space Exploration Group, Huntsville, AL 35898, USA
[5]School of Materials Science and Engineering, Georgia Institute of Technology, Atlanta, GA 30332, USA
**corresponding author, blair.brettmann@chbe.gatech.edu*

A. Shear-thinning profiles

With a high content of solid particles, it can be difficult to maintain continuous flow through the printing nozzle. Looking at the ink's flow behavior through its viscosity response to increasing shear, can provide insights into whether the extrusion will be successful. As a first pass assessment of the suitability of the selected binder formulation in meeting the extrudability criteria set for our ink formulation design, a shear rate sweep was conducted at 25°C on inks containing 65 vol% in the two binders represented in **FIGURE S1**. The shear-thinning behavior is seen in the continuous decrease in viscosity with increasing shear rate in **FIGURE S1**. Because the curve is uninterrupted, even as the shear rates become high, the data indicates that both binder formulations will flow homogeneously upon an applied shear force (for reference, our approximate maximum shear rate during printing is 39 s^{-1}). This shows that regardless of the monomer ratio, both inks are extrudable at 25°C.

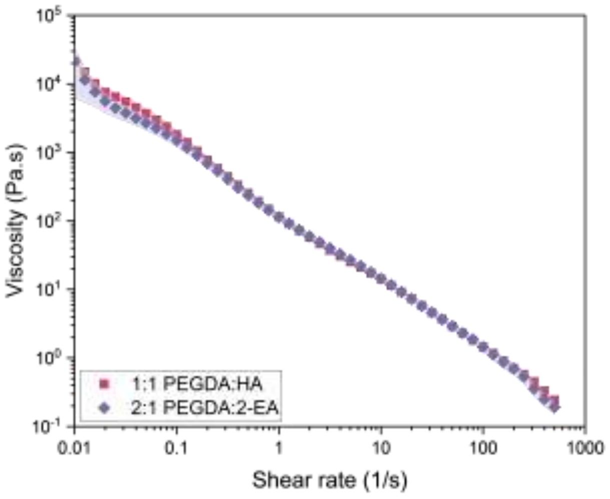

FIGURE S11: Shear-thinning profiles for the 65 vol% 1:1 and 2:1 PEGDA:HA formulations at 25°C. The uninterrupted shear-thinning curves correlate with continuous extrusion of the inks. The apparent shear rate for this printing process is 39s^{-1}. The corresponding viscosities for the 1:1 PEGDA:HA and 2:1 PEGDA:HA formulations are 3.7 Pa.s and 3.6 Pa.s, respectively. The data is averaged across 3 samples with the standard error represented at the shaded region around each curve.

B. Pre and post-curing crystalline phase identification through DSC

In **FIGURE S2A-B**, we are assessing the microstructure of the binder-only samples through a DSC heating cycle both before and after curing with the UV light. As seen in **FIGURE S2A**, prior to UV exposure, all samples containing PEGDA show a crystal melting peak between -20°C and 40°C. After UV exposure however, in **FIGURE S2B**, the crystal melting peak disappears for all formulations except the PEGDA-only. This suggests that the presence of the HA monomer, and its high reactivity at -10°C compared to PEGDA, may result in the PEGDA chains regaining an amorphous state during UV exposure. We hypothesize that initially, HA vinyl bonds are converting first, and that the heat produced from polymerization is sufficient to melt the PEGDA crystalline phase. The amorphous PEGDA chains subsequently crosslink. In the absence of the HA monomer, crosslinking occurs amongst PEGDA chains despite the crystalline phase, but not

enough heat is produced to completely return PEGDA to a fully amorphous state. Therefore, some crystalline domains are still present for the PEGDA-only samples cured at -10°C.

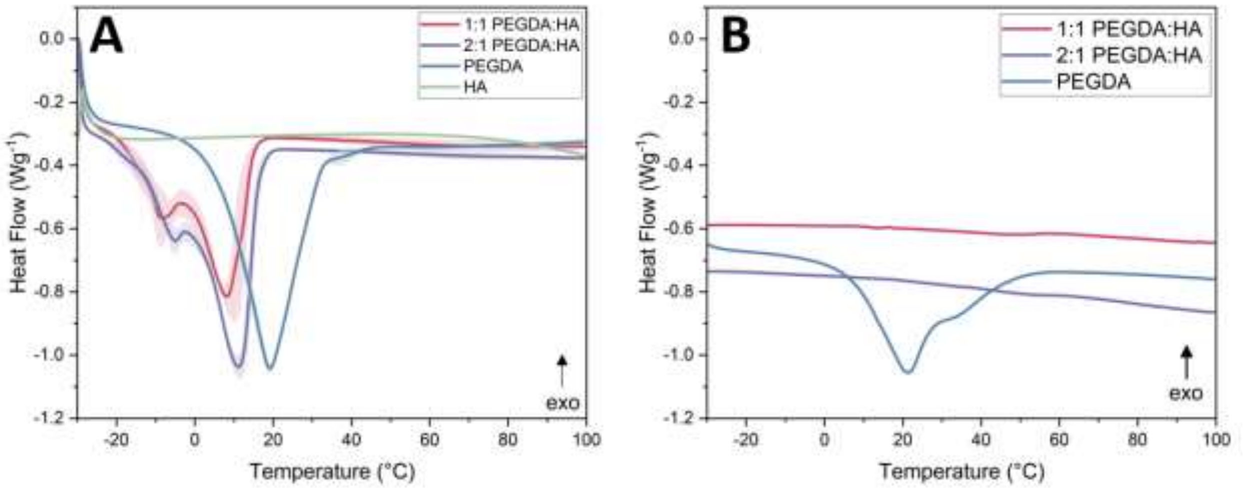

FIGURE S22: A) DSC heating runs on the uncured samples showing an endothermic peak as the PEGDA crystalline phases are melting between -20°C and 40°C. B) DSC heating runs on the cured samples, showing the disappearance of the crystalline melting phase for the 1:1 and 2:1 PEGDA:HA mixtures. The curves plotted are an average of 3 runs, with the standard error across the 3 runs represented as the shaded areas.

<u>C. X-ray Diffraction verifying presence and absence of crystalline domains in cured parts</u>

Figure S3 confirms the absence of crystalline domains in both PEGDA:HA mixtures cured at -10°C, as seen in **FIGURE S2B**. The crystalline peaks observed for the PEGDA-only sample cured at -10°C are indicated at 18.8° and 23.0° 2Θ around the larger amorphous peak. These peaks are not present when running the 1:1 and 2:1 PEGDA:HA samples cured at -10°C, and only the amorphous peak appears.

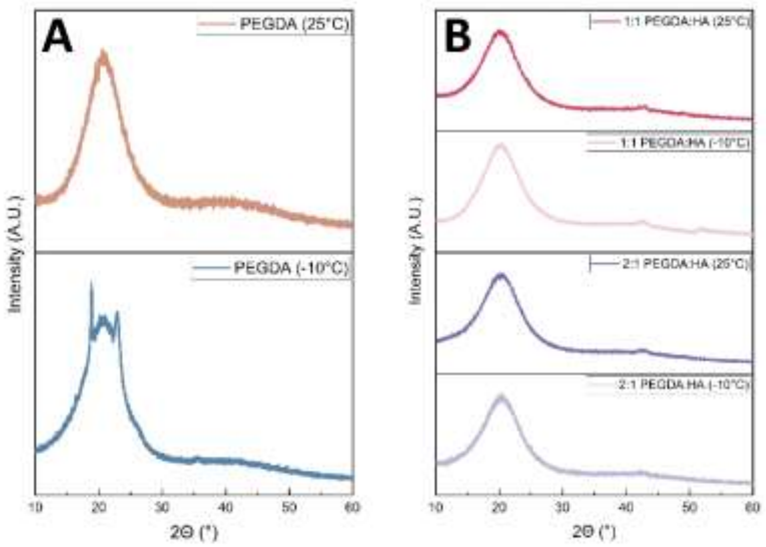

FIGURE S33: A) XRD scans for PEGDA-only cured at both 25°C and -10°C. Notable crystalline peaks appear to either side of the larger amorphous peak when PEGDA is cured at -10°C. The data plotted is the average and standard error of 3 XRD scans. B) XRD

scans on 1:1 and 2:1 PEGDA:HA mixtures also cured at 25°C and -10°C. In these monomer mixtures, the crystalline peaks do not appear for the samples cured at -10°C.

D. Mass loss after swelling

To further verify the hypothesis of more small copolymers and linear HA chains occurring at -10°C, we attempted to quantify the mass loss from both the binder-only and particle-containing samples after swelling. Since we had selected chloroform as the swelling solvent, we could solubilize the small copolymers and HA chains that were not covalently attached to the larger crosslinked network. We hypothesized that, after removal of the solvent and drying of the swollen samples, a change in mass of the samples could be attributed to the removal of these small copolymers and HA chains during solvent extraction. Shown in **FIGURE S4**, all samples lose some amount of their initial mass, but this mass loss is more prominent in 1:1 PEGDA:HA and even more so for the 1:1 PEGDA:HA cured at -10°C. Therefore, provided the mass loss is indeed attributed to the small copolymers and HA chains, we can assume that there is a greater extent of formation of these species at the low temperature in the 1:1 PEGDA:HA ink. This agrees with the data in **FIGURE S5A-C**.

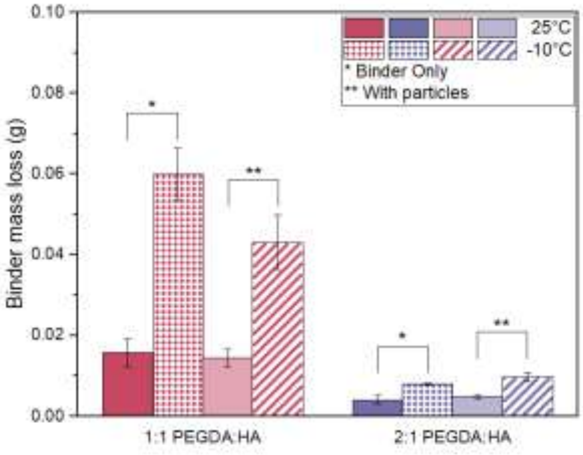

FIGURE S44: Mass loss in binder-only (α) and particle-containing (β) inks of 1:1 PEGDA:HA and 2:1 PEGDA:HA mixtures cured at 25°C (solid fill) and -10°C (patterned fill). The mass loss was obtained as the difference between the starting mass of the samples prior to the swelling experiment and the mass of the samples after swelling, solvent removal, and drying at 60°C for 24 h. The data represented is the mean of 3 samples, with the error bars plotting the standard error across the 3 samples.

E. Effect of post-curing on the depth of cure

For tensile measurements, unreacted monomers are undesirable, since they can skew the deformation mechanics under tensile stress and provide inaccurate results. To minimize the amount of unreacted monomers, especially in the samples printed at cold temperatures, we opted to add a 1-minute post-cure step in a commercially-available curing box at 25°C (these boxes are traditionally used to finish stereolithography/digital light processing 3D printed parts). The UV intensity in the cure box is typically much higher, which can penetrate to greater depths inside the sample prints and initiate curing of

unreacted monomers. In **FIGURE S5A-B**, we have shown how the cure depth of our samples increases drastically after the post-cure. With this large change in cure depths, we expect that most, if not all, of the unreacted monomers for the printed parts would be cured during this step (as a reference, the finished printed parts are only 3 mm tall). Therefore, we expect our tensile testing results to reflect the crosslinking density of the formulations cured during printing, and to not be skewed by unreacted monomers.

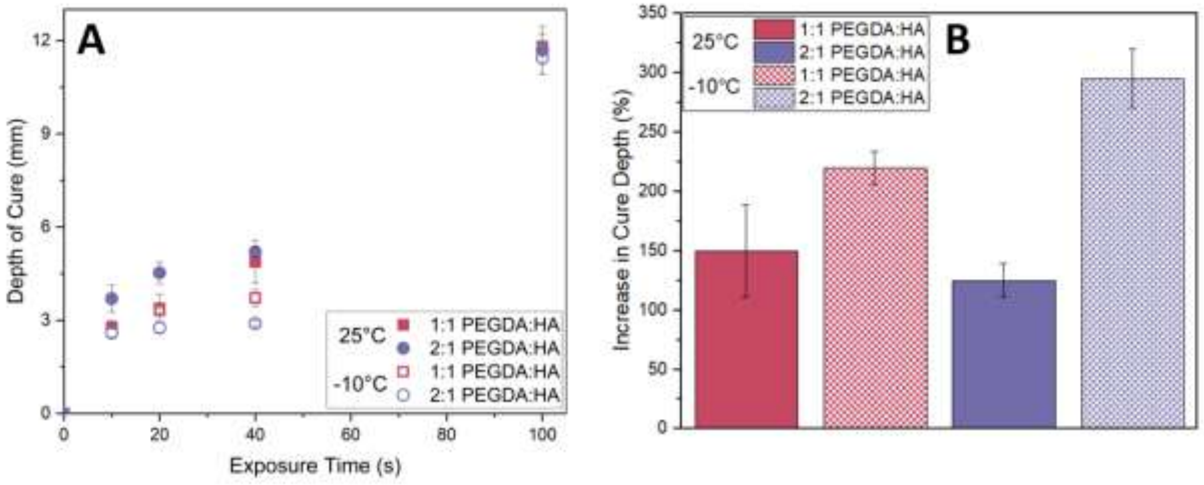

FIGURE S55: Depth of cure after the 1-minute post-cure step (at high intensity in the dedicated cure box) for inks of 65 vol% particles in the 1:1 PEGDA:HA and 2:1 PEGDA:HA binders cured at 25°C and -10°C. A) The post-cured depth of cure is plotted at 100 s on the graph from ***FIGURE 3*** *as a comparison. B) The percent increase in cure depth resulting from the post-cure step compared to the depth of cure recorded at 40 s, showing how the post-cure step greatly improves the depth of cure and minimizes the amounts of monomers still uncured after 40 s of UV exposure. Each data point/bar presented is the average of 3 samples and is plotted with the corresponding standard error.*

F. Acrylate group calculation in both binder formulations

$HA\ M_w = 156.2\ g/mol \qquad HA\ density = 0.88\ g/mL$

$PEGDA\ M_w = 700\ \ g/mol \qquad PEGDA\ density = 1.12\ g/mL$

1) Conversion to molar volume:

$$HA\ M_{vol} = {}^{156.2\ g/mol}\big/_{0.88\ g/mL} = 177.5\ mL/mol$$

$$PEGDA\ M_{vol} = {}^{700\ g/mol}\big/_{1.12\ g/mL} = 625\ mL/mol$$

2) Computation with acrylate groups and volumetric ratios:

- 1:1 PEGDA:HA

$$HA \rightarrow \left(\frac{1\ mL}{177.5\ mL/mol}\right) \times \left(\frac{6.022 \times 10^{23}\ HA\ unit}{1\ mol\ HA}\right) \times \left(\frac{1\ acrylate\ group}{1\ HA\ unit}\right)$$
$$= 3.4 \times 10^{21}\ HA\ acrylate\ groups$$

$$PEGDA \rightarrow \left(\frac{1\ mL}{625\ mL/mol}\right) \times \left(\frac{6.022 \times 10^{23}\ PEGDA\ unit}{1\ mol\ PEGDA}\right) \times \left(\frac{2\ acrylate\ group}{1\ PEGDA\ unit}\right)$$
$$= 9.64 \times 10^{20}\ HA\ acrylate\ groups$$

$$\mathbf{1:1\ PEGDA:HA\ total\ number\ of\ acrylate\ groups = 5.32 \times 10^{21}}$$

- 2:1 PEGDA:HA

$$HA \rightarrow \left(\frac{1\ mL}{177.5\ mL/mol}\right) \times \left(\frac{6.022 \times 10^{23}\ HA\ unit}{1\ mol\ HA}\right) \times \left(\frac{1\ acrylate\ group}{1\ HA\ unit}\right)$$
$$= 3.4 \times 10^{21}\ HA\ acrylate\ groups$$

$$PEGDA \rightarrow \left(\frac{2\ mL}{625\ mL/mol}\right) \times \left(\frac{6.022 \times 10^{23}\ PEGDA\ unit}{1\ mol\ PEGDA}\right) \times \left(\frac{2\ acrylate\ group}{1\ PEGDA\ unit}\right)$$
$$= 1.93 \times 10^{21}\ HA\ acrylate\ groups$$

$$\mathbf{2:1\ PEGDA:HA\ total\ number\ of\ acrylate\ groups = 7.25 \times 10^{21}}$$
